# Nanostructured copper molybdates as promising bifunctional electrocatalysts for overall water splitting and CO_2_ reduction†

**DOI:** 10.1039/d0ra07783f

**Published:** 2020-10-23

**Authors:** Atefeh Rahmani, Hossein Farsi

**Affiliations:** Department of Chemistry, University of Birjand Birjand Iran hofarsi@birjand.ac.ir; Developing Nanomaterials for Environmental Protection Research Lab, University of Birjand Birjand Iran

## Abstract

Overall water splitting and CO_2_ reduction are two very important reactions from the environmental viewpoint. The former produces hydrogen as a clean fuel and the latter decreases the amount of CO_2_ emissions and thus reduces greenhouse effects. Here, we prepare two types of copper molybdate, CuMoO_4_ and Cu_3_Mo_2_O_9_, and electrochemically investigate them for water splitting and CO_2_ reduction. Our findings show that Cu_3_Mo_2_O_9_ is a better electrocatalyst for full water splitting compared to CuMoO_4_. It provides overpotentials, which are smaller than the overpotentials of CuMoO_4_ by around 0.14 V at a current density of 1 mA cm^−2^ and 0.10 V at −0.4 mA cm^−2^, for water oxidation and hydrogen evolution reactions, respectively. However, CuMoO_4_ adsorbs CO_2_ and the reduced intermediates/products more strongly than Cu_3_Mo_2_O_9_. Such different behaviors of these electrocatalysts can be attributed to their different unit cells.

## Abstract 2

Comparing overall water splitting on the surface two types of copper molybdate.

## Graphical abstract

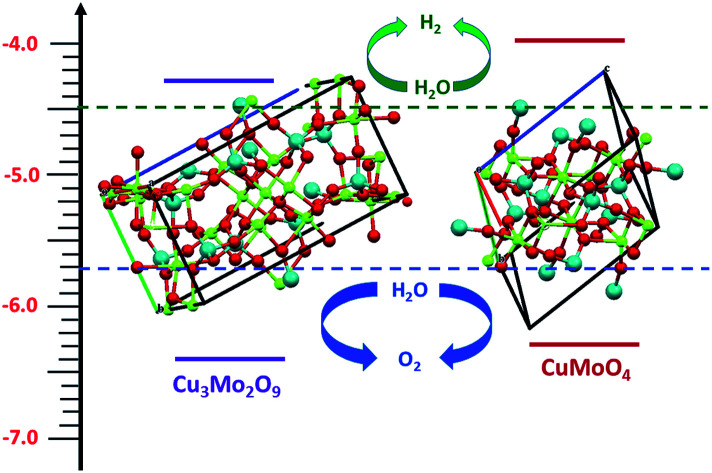

## 1. Introduction

Fossil fuels are diminishing resources that contribute notably to environmental pollution, so developing sustainable energy sources suitable for our growing energy requirements is critical for the survival of humankind.^1,2^ An abiotic component of promising renewable energy infrastructure with no reliance on fossil fuels and no carbon dioxide emission is the splitting of water into hydrogen and oxygen.^3,4^ In photoelectrochemical (PEC) water splitting, solar energy is directly converted to fuel by light-absorbing semiconductors. As a new generation energy carrier, solar hydrogen will play an important role in our lives because it is storable, transportable, and convertible into efficient electricity in fuel cells, as well as being a viable and clean source of power due to its extraordinarily high energy density.^5^ During water splitting, the reductive half-reaction with a two-electron transfer mechanism is relatively easier than the oxidative half-reaction involving a four-electron transfer mechanism,^6^ which is the most energy-intensive step in the overall water splitting process. Therefore, the slow kinetics of the oxygen evolution reaction, OER, requires a substantial overpotential to generate measurable current densities.^7^ A large positive change in Gibbs free energy (Δ*G*^0^ = 237.13 kJ mol^−1^) causes the full water splitting reaction to be thermodynamically an uphill reaction.^8^ From this perspective, it is challenging to design highly effective photocatalysts while considering crucial parameters in the development of suitable semiconductors. These parameters include band gap width, optical absorption edge, stability against photocorrosion/lifetime, solar photons to current efficiency, cost-effectiveness, catalytic activity and surface structure.^9–11^ Furthermore, a high degree of crystallinity, *i.e.* a smaller amount of defects, is often required for water splitting because recombination between photogenerated carriers is particularly a serious problem in uphill reactions. Beginning with the discovery of Fujishima and Honda in the early 1970s, metal oxide or mixed-metal oxide semiconductors have been well studied as water splitting photoanodes due to their low cost and high stability in aqueous environments.^12^ Because the mixture of two metals in an oxide matrix can generate materials with unique chemical and physical properties, much consideration has been paid to the mixed-metal oxides, such as metal titanates, metal tungstates and metal molybdates.

Moreover, the growing CO_2_ emission is an inevitable result of fossil fuels consumption because of the accessibility, diversity and high energy density of these fuels. However, the ever-increasing use of fossil fuels will cause dependence on them despite their limited reserves; additionally, global warming, drastic environmental changes and an intense threat to human survival are other issues associated with the use of fossil fuels. To resolve these issues, the best approach is to convert CO_2_ into carbon-containing fuels as a renewable energy resource.^13,14^ Recently, electrochemical reduction of accumulative CO_2_ has been considered as a feasible strategy for the conversion and utilization of this gas. This process was reported for the first time by Sir B. C. Brodie in 1873 and later in the 1990s by Hori *et al.*^15,16^ However, many efforts have been carried out to overcome usual challenges, such as poor efficiency because of kinetic barrier for stable molecules, weak selectivity resulting from reduction products competition, side reactions and hydrogen evolution reaction (HER).^17,18^ Therefore, probing new electrocatalysts that efficiently reduce CO_2_ into liquid fuels, especially in mild conditions, is extremely significant. For this purpose, copper (Cu) and Cu-based electrodes have been investigated as electrocatalyst candidates due to their intrinsic catalytic activity and variation of reduction products, which considerably depend on surface properties and morphology.^19–30^ Despite many experimental efforts and computational calculations, the mechanism of CO_2_ reduction on Cu electrodes is intricate and the exact reaction path is controversial.^31,32^

With regard to the above studies, it seems that Cu-based mixed oxides can be considered as bifunctional-electrocatalysts, which can electrochemically oxidize water and reduce CO_2_. For example, some of the widely studied copper-based photocatalysts for water splitting have been crystalline copper phosphide nanosheet,^33^ cuprous oxide composites,^34–36^ copper(ii) tungstate^37–39^ and copper(ii) borate.^40^ More recently, scientists have tried to find nanomaterials that exhibit remarkable properties to catalyze both hydrogen production and water oxidation reactions when the applied potentials fluctuate between oxidative and reductive conditions in the same reaction electrolyte.^41^ A copper-based catalyst composite film was found to be an interesting electrocatalyst for both HER and OER in the same electrolyte.^42^

To the best of our knowledge, there is no report on the application of copper molybdates for these properties. However, over the past several years, different preparations and structures of transition metal molybdates have been widely reported; these include doped,^43,44^ layered,^45–47^ nanosheet,^48,49^ powder,^50^ flower-like,^51^ nanoplate^52^ and micropompons^53^ structures. These structures are widely used in the areas of catalysis, magnetic application and energy storage. Furthermore, CuMoO_4_ is a thermochromic, trisochromic and piezochromic material, so it can be used in pressure and/or temperature sensors. Recently, extensive studies have been conducted on using transition metal molybdates in catalysis/photocatalysis,^43,44,49,53–55^ magnetic applications^46,56–58^ and lithium batteries;^52^ for example, preparation of hybrid Cu_2_O/CuMoO_4_ nanosheet electrodes for high-performance asymmetric supercapacitors,^48^ Cu_3_Mo_2_O_9_ nanoplates with excellent lithium storage performance^52^ and Cu_3_Mo_2_O_9_ micropompons with excellent performance in photocatalytic degradation of Congo red under visible light, photocurrent response and lithium storage.^53^ Two different compounds of copper molybdate exist with two different chemical formulae of CuMoO_4_ and Cu_3_Mo_2_O_9_. Both of them contain MoO_4_ tetrahedra, CuO_6_ octahedra and CuO_5_ pyramid units. In CuMoO_4_, six copper–oxygen polyhedra share edges to form a spiral-shaped chain fragment, interconnected by MoO_4_ tetrahedra,^59^ whereas sharing the edge oxygen atoms of Cu-based polyhedra makes a zigzag ribbon.^60^

**Fig. 1:**
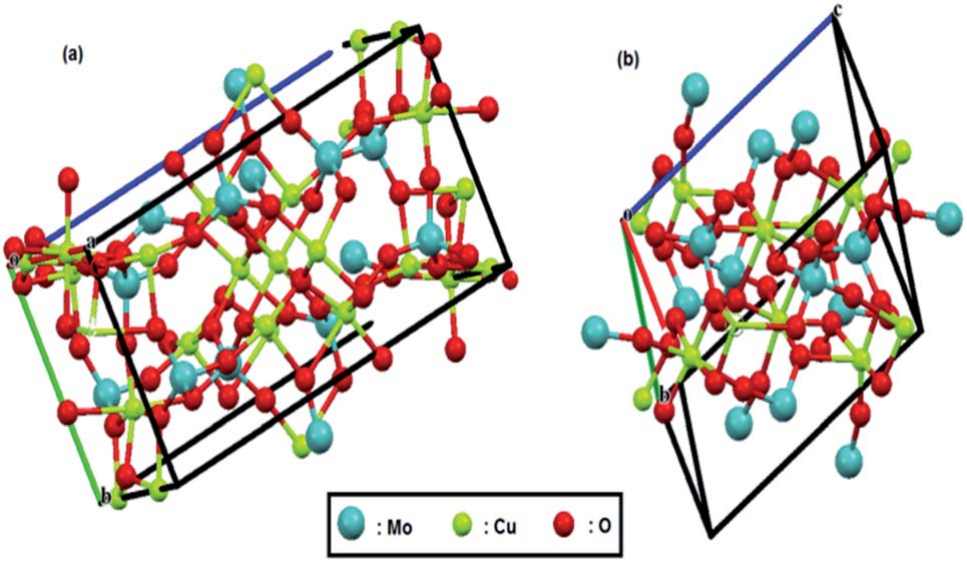
Crystal structure of (a) Cu_3_Mo_2_O_9_ and (b) CuMoO_4_. Red, green and pale blue balls represent oxygen, copper and molybdenum atoms, respectively.

The crystal structures of both compounds are shown in Fig. 1; these structures were reproduced by Mercury software^61–63^ from the Cambridge Crystallographic Data Center, using their CIF files. Recently, we reported that tungstate-derived copper has a good activity for electrochemical reduction of CO_2_,^64^ so it is expected here that we have molybdate-derived copper from two different sources, CuMoO_4_ and Cu_3_Mo_2_O_9_, with two different distribution of copper atoms and consequently different activities.

Due to these different crystal structures, their comparative study as bifunctional catalysts for electrochemical water splitting and CO_2_ reduction seems very interesting. In this study, we successfully prepare, characterize and electrochemically investigate both nano-CuMoO_4_ and nano-Cu_3_Mo_2_O_9_ structures.

## 2. Experimental

### 2.1. Materials preparation

The reactions were carried out under air at room pressure. High purity (>99%) AR grade copper nitrate Cu(NO_3_)_2_·3H_2_O, sodium molybdate Na_2_MoO_4_·2H_2_O, citric acid and ammonium hepta-molybdate, (NH_4_)_6_Mo_7_O_24_·4H_2_O, were obtained from commercial sources.

For the synthesis of Cu_3_Mo_2_O_9_, 2.4195 g (0.01 mmol) Na_2_MoO_4_·2H_2_O was dissolved in 50 mL deionized water to form a transparent solution. Then, 2.4160 g (0.01 mmol) Cu(NO_3_)_2_·3H_2_O was dissolved in 10 mL deionized water to reach a dark blue solution, which was added to the first solution dropwise by a burette and stirred at room temperature for 30 min. After stirring, the beaker was subjected to 80 W cm^−2^ ultrasonic radiation under air for 30 min using an ultra sonicator operating at 40 kHz. The reaction was carried out in a glass balloon, which was heated in an oil bath at about 100 °C for 20 h. The balloon was connected to a reflux condenser system open to the atmosphere. Next, the resulted particles were filtered and washed several times with distilled water and ethanol and then dried in an oven at a temperature of 75 °C for 3 h. The obtained powders were treated under air at temperatures ranging from 25 °C to 400 °C for 2 h and were then remained at this temperature for 4 h. Finally, Cu_3_Mo_2_O_9_ was prepared as a brown-orange powder.

**Fig. 2:**
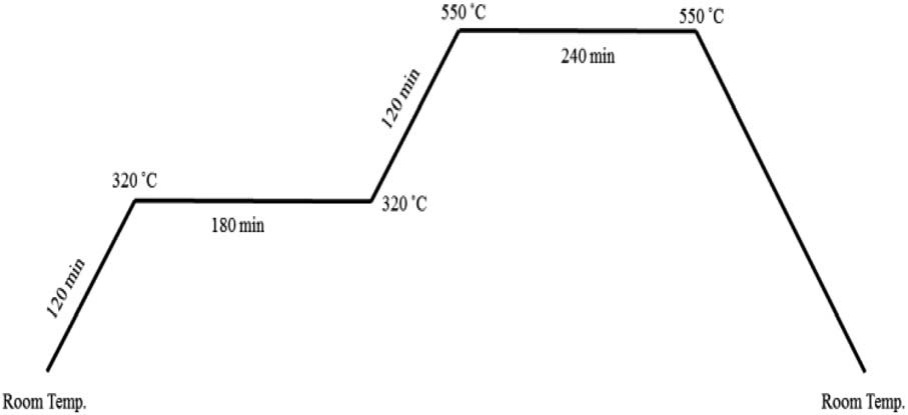
Temperature profile for calcination process.

For the preparation of CuMoO_4_, 5 mL of copper nitrate solution (0.50 M) was added to molybdate solution, which was obtained by dissolving 0.4414 g ammonium heptamolybdate in 50 mL deionized water (pH = 4.7). The mixture was stirred about 15 min, and then a citric acid (CA) solution in a molar proportion of 3 : 1 CA : cation was added. By evaporating the homogeneous solution at 90 °C (low-temperature crystallization), a sky blue gel was gradually formed. The obtained gel was transferred to a highly porous Prussian blue matrix at 120 °C for 24 h; then, it was calcined over a wide range of temperatures and times as shown in Fig. 2 to optimize its surface area, crystallinity and photocatalytic activity.^64^ Finally, a green yellowish powder that was obtained at the end of the process was characterized.

### 2.2. Electrode fabrication

For electrochemical studies, working electrodes were prepared by the electrophoretic method. Two 1 × 5 cm^2^ pieces of commercial stainless steel with a separation distance of 2 cm were inserted into a mixture, which was prepared by dispersing 0.0056 g of the prepared samples in 5 mL ethanol and five-minute exposure under 50 kHz ultrasonic waves. A potential of 20 V was applied between the two electrodes for 60 s. Next, the prepared electrodes were dried at 80 °C for 3 h and finally at 400 °C for 15 min.

### 2.3. Material characterization

XRD pattern of CuMoO_4_ and Cu_3_Mo_2_O_9_ samples were recorded on an XPERT-PRO powder X-ray diffractometer. The CuKα (*k* = 1.5406 and 1.5443 Å) radiation was used, and diffractions were measured over 2*θ* range of 5–80°. Transmission electron microscopy (TEM) images were obtained with a CM120 electron microscope from Philips. The diffuse reflectance spectra were determined with a UV-Vis spectrophotometer Avantes (Avaspec-2048-TEC) with BaSO_4_ as the standard reference. The magnetic properties were measured using a vibrating sample magnetometer (VSM) system (Lake Shore7404).

### 2.4. Electrochemical characterization

All electrochemical measurements on the surface of catalysts were conducted in a 0.1 M NaOH (pH = 13) solution by a standard three-electrode cell containing Ag/AgCl as the reference electrode, a platinum mesh as the auxiliary electrode and an electrophoretically deposited material electrode as the working electrode using a Solartron SI-1260 electrochemical interface. For Mott–Schottky analysis, a Solartron Phase Gain Analyzer SI1260 was used to determine electrochemical impedance spectra (EIS) by handling a frequency of 10 000 Hz, an AC voltage amplitude of 10 mV in different scanning potential ranges for the deposited electrode *vs.* Ag/AgCl (between −0.35 and 0.2 V for CuMoO_4_, and between −0.45 and 0.4 V for Cu_3_Mo_2_O_9_) both in the dark and under illumination by a fluorescent lamp. A range of frequencies between 0.1 and 100 000 Hz was used in EIS measurements with a 10 mV amplitude perturbation.

The electrocatalytic activities of the working electrodes were tested using Cyclic Voltammetry (CV) and Linear Sweep Voltammetry (LSV), at a scan rate of 10 mV s^−1^ in different potential ranges. All electrochemical tests were performed at ambient temperature and pressure. To reduce the additional ohmic resistance, bubbles that were generated on the electrode surface under OER and HER were dispersed by stirring the solution at 1000 rpm during the reaction. Moreover, to enhance the mass transport of CO_2_ during the electroreduction, CO_2_ was continuously bubbled.

## 3. Results and discussion

### 3.1. XRD analysis

**Fig. 3:**
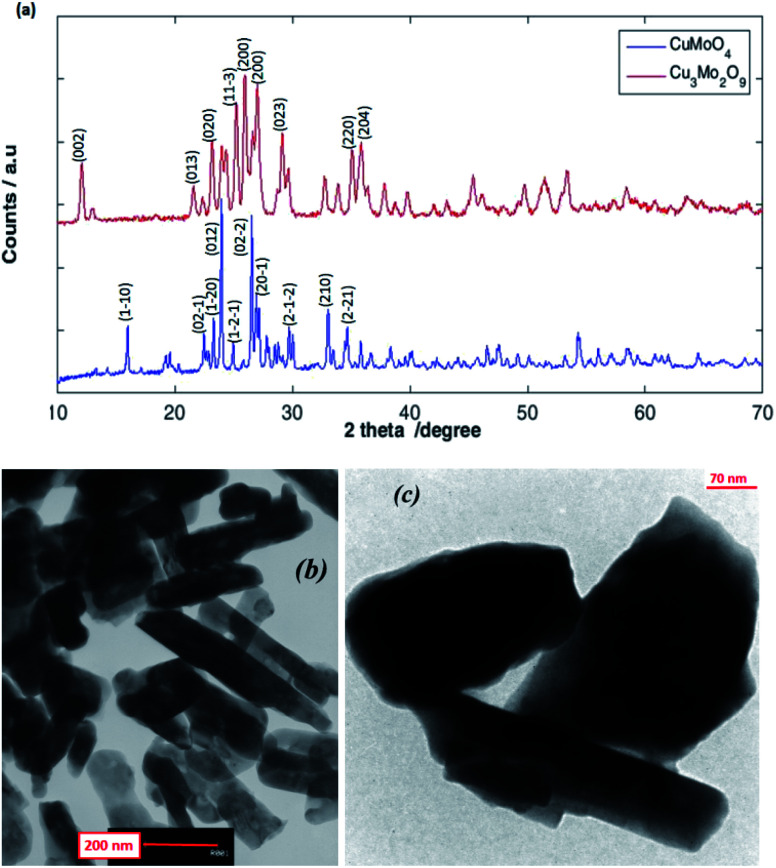
(a) XRD patterns, and TEM images for (b) Cu_3_Mo_2_O_9_ and (c) CuMoO_4_.

As shown in Fig. 3a, the XRD patterns of the as-prepared CuMoO_4_ and Cu_3_Mo_2_O_9_ samples reveal their crystalline nature, which are consisted of a single-phase and orthorhombic Cu_3_Mo_2_O_9_ with space group *Pna*2_1_ and anorthic CuMoO_4_ with space group *P*1̄. As shown, the broadening of the diffraction peaks proves the high purity as well as the nanocrystalline nature of the as-prepared samples. The strong sharp diffraction peaks indicate that the samples are well crystallized. The peaks can be indexed to orthorhombic Cu_3_Mo_2_O_9_ (JCPDS 94-0728)^60^ and anorthic CuMoO_4_ (JCPDS 98-000-7372).^59^ The strong peaks at the defined 2*θ* values of 25.96, 27.02 and 25.24 can be indexed to (002), (140) and (131) planes of Cu_3_Mo_2_O_9_, and at 2*θ* values of 23.95, 26.53 and 27.16 to (012), (201) and (211) planes of CuMoO_4_ respectively. The (002) and (012) reflection peaks were used for the calculation of the crystallite size of Cu_3_Mo_2_O_9_ and CuMoO_4_, respectively. The average crystallite size calculated using the Debye–Scherrer equation from the FWHM (full-width half maxima) of the XRD peaks of Cu_3_Mo_2_O_9_ and CuMoO_4_ samples were found to be approximately 49 and 134 nm, respectively.

### 3.2. TEM

TEM studies provide further insight into the nanostructure and topography of the treated samples. Fig. 3b and c shows the TEM images of Cu_3_Mo_2_O_9_ and CuMoO_4_ for the formation of rod-shaped orthorhombic and multiform shape particles of the samples; both monocrystalline particles have diameters that are in good agreement with the size obtained from powder XRD. The nanoparticles are a little agglomerated.

### 3.3. Magnetic properties analysis

**Fig. 4:**
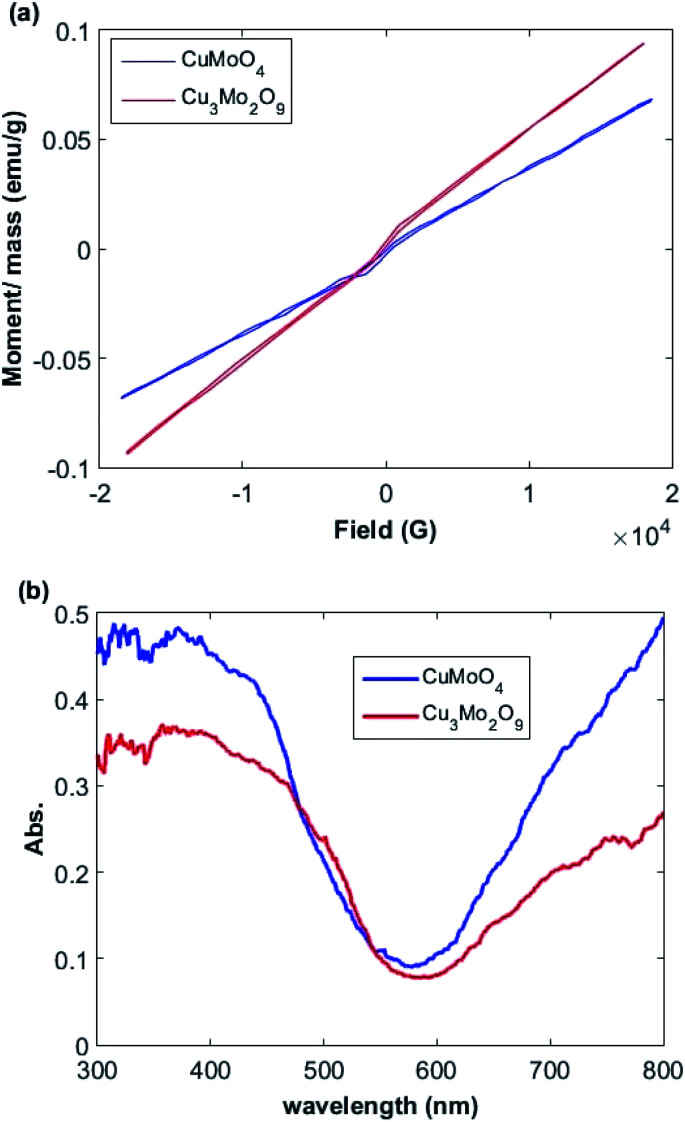
(a) Magnetization curves and (b) UV-Vis diffuse reflectance spectra of Cu_3_Mo_2_O_9_ and CuMoO_4_.

The relationships between the magnetization (*M*) and magnetic field (*F*) of Cu_3_Mo_2_O_9_ and CuMoO_4_ nanoparticles was determined by a vibrating sample magnetometer (VSM) system (Lake Shore 7404). Fig. 4a shows the M–F curve in the range from −18 to 18 kG with a saturated magnetization. A linear increment above 7 kG with a narrow hysteresis loop occurs between −2 and 2 kG; this pattern is the characteristic of both ferromagnetic and antiferromagnetic interactions for both samples.^65,66^

### 3.4. Optical properties

The optical properties of the samples were measured by UV-Vis DRS spectrum. Fig. 4b demonstrates the DRS spectra of the prepared Cu_3_Mo_2_O_9_ and CuMoO_4_ samples. Two sequential absorption bands can be identified in these samples. One band is located above 600 nm, and the other one below 600 nm; these results are in good agreement with the color of the samples. The former can be mainly attributed to d–d transition of Cu^2+^ ions (3d^9^), whereas the latter may be due to the Cu(3d) ← O(2p) LMCT or Mo(4d) ← O(2p) LMCT and an Mo(4d) ← Cu(3d) MMCT, which is located at wavelengths higher than LMCT band.^66,67^

The direct and indirect optical band gaps of nanoparticles were extracted according to Tauc's equation.^68,69^ According to Tauc's equation, the relation between the absorption coefficient (*α*) and the incident photon energy (*hν*) can be written as:

1 (*αhν*)^1/*n*^ = *A*(*hν* − *E*_g_)

where *A* is a constant, *E*_g_ is the band gap energy of the materials; the exponent *n* depends on the transition type and is equal to 0.5 and 2 for direct and indirect transitions, respectively.^70^ To determine the possible transitions, (*αhν*)^1/*n*^ was plotted *versus hν*, and the corresponding band gaps were obtained by extrapolating the steepest part of the graph on the *hν* axis for (*αhν*)^1/*n*^ = 0. The direct and indirect band gap energies were obtained from, respectively, (*αhν*)^2^ and (*αhν*)^1/2^*vs. hν* as shown in Fig. S1.†

The larger band gaps of both samples correspond to the direct electronic transition between the upper edge of O:2p valence band and the lower edge of Mo:4d conduction band. The calculated band gaps for these transitions are 2.4 and 2.6 eV for Cu_3_Mo_2_O_9_ and CuMoO_4_, respectively. The lower energy indirect transitions can be explained by assuming the existence of narrow band correlated-electron states in the band gap instead of a single wide band of uncorrelated-electron states that may correspond to Cu^2+^:3d^9^ band. We speculate that in addition to the contribution of Cu (3d) on the top of O (2p) valence band, the conduction band of both samples contain Cu (3d) characteristic, in agreement with our experimental observations. The Jahn–Teller distortion of Cu^2+^ gives rise to a d-orbital splitting in which the degeneracy of σ-antibonding orbitals is broken.^38^ The calculated Tauc's plot indirect band gaps are 2.1 and 2.3 eV for Cu_3_Mo_2_O_9_ and CuMoO_4_ samples, respectively, so their absorption edges are in the visible range of the spectrum. Ansari *et al.* reported that the electronic transitions of CuMoO_4_ are of indirect type and a band gap of 3.06 eV was obtained,^71^ whereas values of 2.38, 2.80 and 2.15 eVs were obtained by other researchers.^72–74^ Moreover, Dutta *et al.* found an indirect band gap of 2.32 eV.^54^

### 3.5. Mott–Schottky analysis

For Mott–Schottky analysis, one has to select a range of potentials in which Faraday's reaction does not occur. To determine this range, cyclic voltammetric technique was used. The characteristic of Faraday's processes is the evolution of peaks in cyclic voltammograms. For non-faradic or capacitive processes, no peak is observed and the voltammogram is almost rectangular. Cyclic voltammograms with a scan rate of 10 mV s^−1^ in potential ranges between 0.2 and −0.35 for CuMoO_4_ and between 0.25 and −0.45 V for Cu_3_Mo_2_O_9_ in 0.1 M NaOH were obtained. In this way, a range of potentials was determined over which the cyclic voltammograms showed no faradic current, and only a capacitive current was observed. In order to ensure that the observed behavior in the voltammograms was capacitive, the effect of different scan rates on the voltammograms was evaluated. If the observed behavior is capacitive, the anodic and cathodic currents should increase with increasing the scan rate. Therefore, the performance of samples in these potential regions can be considered as capacitive, as shown in Fig. S2.†

**Fig. 5:**
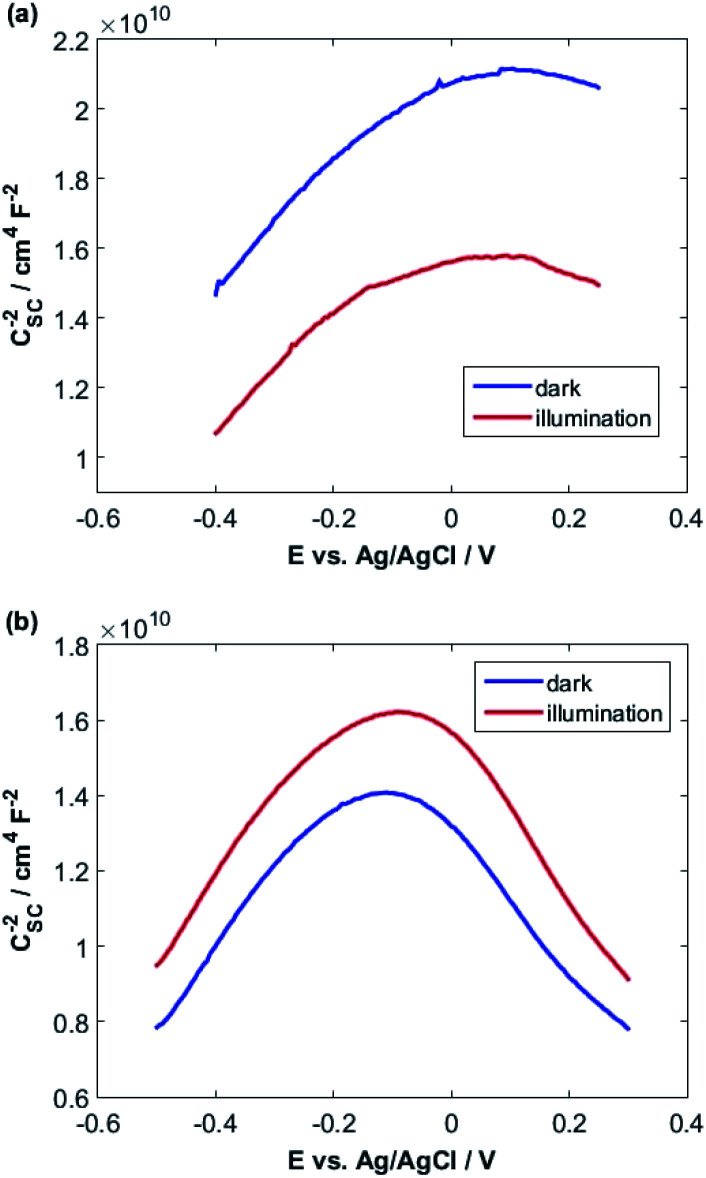
Mott–Schottky plots both in the dark and under illumination for (a) CuMoO_4_ and (b) Cu_3_Mo_2_O_9_.

When a semiconductor is exposed to an electrolyte, it reaches the thermodynamic equilibrium with the electrolyte by exchanging electrons through the interface and adjusting the Fermi level of the semiconductor to the Fermi level of the electrolyte, which results in the formation of a space charge layer. The Fermi level position of the electrolyte is constant because the number of available states in the electrolyte solution is typically more than the number of states in the semiconductor. With regard to electron transfer from/to a semiconductor to/from an electrolyte, an excess of immobilized charge in the semiconductor and an excess of opposite charge in the electrolyte are produced just behind the interface and in the space charge layer. This implies that the energy bands are bent,^75^ and the amount of bending depends on the difference between band edge energy in the semiconductor and at the interface; furthermore, the amount of bending corresponds to the potential drop in the space charge layer, *U*_S_. When an external electrical source is applied, then *U*_S_ = *U*_app_ − *U*_FB_, where *U*_FB_ is the flat band potential, that is, a unique potential for which the potential drop between the bulk and surface is zero. Thus, by determining the flat band potential one can estimate the situations of conduction and valence band edges for n-type and p-type semiconductors, respectively.^76^ Mott–Schottky theory describes the properties of semiconductor electrolyte interface using space charge capacitance as follows^77–79^2
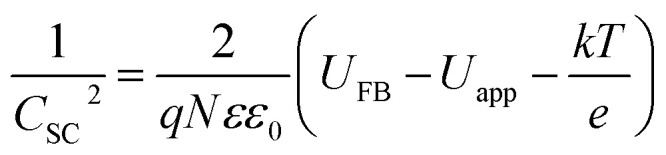
where *ε* is the relative permittivity of the semiconductor, *ε*_0_ is the vacuum permittivity and is equal to 8.85 × 10^−12^ F m^−1^; *q* is the elementary charge (−1.6 × 10^−19^ C for electrons and +1.6 × 10^−19^ for holes), *N* is the carrier charge concentration, *U*_app_ is the applied potential, *U*_FB_ is the flat band potential, *T* is the absolute temperature and *k* is the Boltzmann's constant and is equal to 1.381 × 10^−23^ J K^−1^. The latest term in eqn (2), *kT*/*e* = 0.026 V at 300 K, is small and can be omitted. CSC can be calculated from impedance as, *C*_SC_= (−1)/(2π*νZ*_j_) where *ν* is the frequency and *Z*_j_ is the imaginary part of the impedance. Fig. 5a and b shows Mott–Schottky plots for both samples at a frequency of 10 000 Hz in 0.1 M NaOH in the dark and under illumination.

A fairly large frequency of 10 000 Hz is chosen since fast processes such as charging of double layer and space charge layer capacitors respond at this frequency. Obviously, selecting and recognizing the linear region are difficult. Therefore, since at this frequency, the samples exhibit capacitive behavior and the capacity of the space charge layer is independent of potential, the Nyquist diagrams are in a range of potentials selected from the CVs. It is expected that these diagrams will coincide almost exactly with the region where only the space layer charging occurs.

For this purpose, Nyquist diagrams with 0.05 V potential interval and over a frequency range of 0.1 to 100 000 Hz are plotted in the dark and under illumination in 0.1 M NaOH solution (Fig. S3 and S4†). As illustrated in these figures, Nyquist diagrams over the range −0.24 to −0.12 V for CuMoO_4_ and the range −0.30 to −0.18 V for Cu_3_Mo_2_O_9_ in the dark are fairly in good agreement. Therefore, these regions were used for the linear fitting of the Mott–Schottky equation, and the results are shown in Fig. S5 and S6.† A correlation coefficient close to 1 implies a good agreement between the experimental results and the results of the Mott–Schottky theory. The value of *U*_FB_ can be determined from the intercept of the fitting line with the Mott–Schottky plot. In Fig. S5 and S6,† the slopes of Mott–Schottky plots for both electrodes are positive; this confirms that the electrodes are n-type semiconductors, and consequently, the lower conduction band edge practically merges with the quasi-Fermi level of electrons. The conduction band edge, *E*_C_, can be experimentally estimated using flat band potential (*U*_FB_).^80^ For this propose, the electrochemical potentials should be related to absolute energy, *E*_abs_, and the effects of pH should be considered using the following equations at 25 °C:

3 *E*_RHE_ (V) = *E*_Ag/AgCl_ + 0.199 + 0.0592pH

4 *E*_abs_ (eV) = −4.5 − *E*_RHE_

where *E*_RHE_ and *E*_Ag/AgCl_ are potentials against the reversible hydrogen electrode and Ag/AgCl reference electrode, respectively.

As can be seen, different values were obtained for the flat band potentials for both samples. The values of *E*_C_ for both samples were determined using the *U*_FB_ values reported in Table 1 and eqn (3) and (4).

**Table 1:** Some calculated parameters using Mott–Schottky analysis for both CuMoO_4_ and Cu_3_Mo_2_O_9_ samples

Sample	Condition	*U*_FB_/V	*N*_D_/cm^−3^	*W*_D_/cm	*L*/cm	*E*_C_ *vs.* NHE/V	*E*_C_ (abs)/eV	*E*_C_ (abs)/eV	*E*_g_/eV
CuMoO_4_	Dark	−1.50	1.25 × 10^21^	8.36 × 10^−8^	9.44 × 10^−9^	−0.53	−4.0	−6.3	2.3
	Illumination	−1.29	1.38 × 10^21^	7.95 × 10^−8^	9.02 × 10^−9^	−0.32	−4.2	−6.5	2.3
Cu_3_Mo_2_O_9_	Dark	−1.18	1.42 × 10^21^	7.48 × 10^−8^	8.49 × 10^−9^	−0.21	−4.3	−6.4	2.1
	Illumination	−1.36	1.47 × 10^21^	7.36 × 10^−8^	8.34 × 10^−9^	−0.39	−4.1	−6.2	2.1

**Fig. 6:**
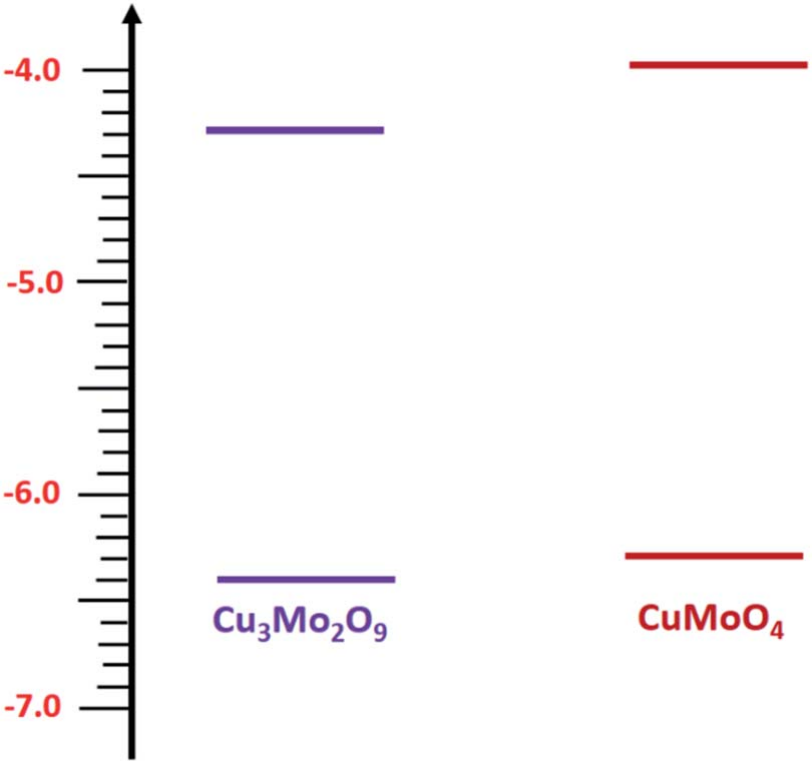
Energy states diagram of CuMoO_4_ and Cu_3_Mo_2_O_9_.

Then, with a method, which has already been described in the electrochemical studies of nanostructured nickel titanate^81^ and benefits from a combination of optical (DRS) and electrochemical (impedance) spectroscopies, we determined the valence band edges, *E*_V_, of both samples with subtracting the values of band gap energies from *E*_C_. All band edges for both samples are reported in Table 1 for in the dark and under illumination. According to these data, we outline the experimental electronic band structures of both samples as illustrated in Fig. 6. The O^2−^:2p and Cu^2+^:3d orbitals contribute to the valence band; additionally, O^2−^:2p and Mo^6+^:4d orbitals contribute to the conduction band for both compounds.^82^

The density of donor atoms can be calculated using the slope value of Mott–Schottky plots (Fig. S5 and S6†) and eqn (2). The calculated values are shown in Table 1. The density of donor atoms, especially copper atoms, in Cu_3_Mo_2_O_9_ is larger than CuMoO_4_. Moreover, using *N*_D_ values, it is possible to estimate the diffusion length of minor charge carriers (here holes) using the following equations at 25 °C:5
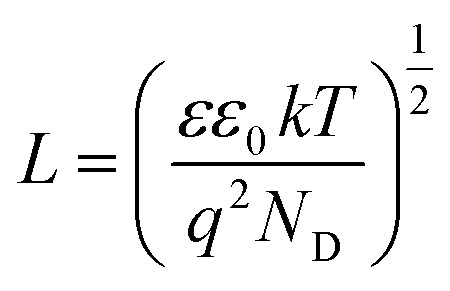
6
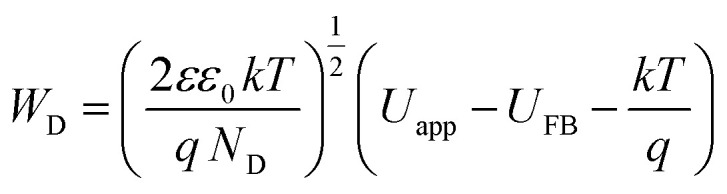


The obtained results are shown in Table 1 considering that the dielectric constants of Cu_3_Mo_2_O_9_ and CuMoO_4_ are equal to 7.2 (ref. 83) and 7.9,^84^ respectively.

One can calculate diffusion length of minority carrier, *L*, and the thickness of the depletion layer, *W*_D_, by using *U*_FB_, *N*_D_ and eqn (2). The values of *N*_D_, *L* and *W*_D_ (for *U*_app_ − *U*_FB_ = 1 V) were computed, and the results (accompanied with *U*_FB_) are listed in Table 1.

### 3.6. Electrochemical water splitting studies

Electrochemical water splitting includes oxygen evolution reaction, OER, on the anode surface and hydrogen evolution reaction, HER, on the cathode surface. Electrochemical water splitting is a pH-dependent reaction, so it has been widely investigated in both acidic and alkaline solutions. From the technological viewpoint, Schalenbach *et al.* showed that alkaline water electrolyzers are more efficient compared to acidic ones.^85^ Moreover, from the electrocatalyst design viewpoint, alkaline solutions allow us to use earth-abundant materials, especially for OER,^86,87^ whereas acidic solutions are limited to precious metals and metal oxides.^88^ However, the big disadvantage of alkaline solutions is the sluggish kinetics of OER in these media.^89^

This is due to the four-electron-proton transfer mechanism in alkaline media, which is the rate-determining step at high overpotentials. Therefore, to compare CuMoO_4_ and Cu_3_Mo_2_O_9_ in electrochemical OERs, one should calculate their potentials and kinetic parameters such as exchange current density and Tafel slope. The overall reaction, which occurs on the surface of the electrocatalyst is:

7 4OH^−^ + 4h_VB_^+^ →2H_2_O + O_2_

**Fig. 7:**
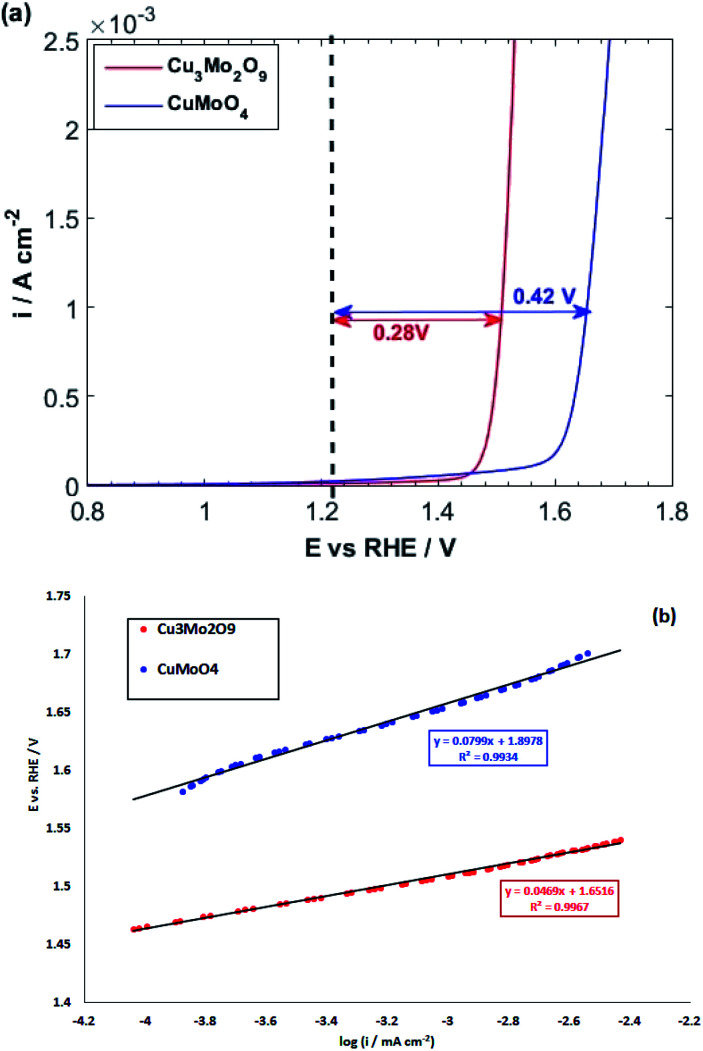
(a) LSVs and (b) linear parts of polarization curves of CuMoO_4_ and Cu_3_Mo_2_O_9_ for OER with a scan rate of 10 mV s^−1^ in 0.1 M NaOH.

During this complex reaction, four electrons are transferred to the valence band of the semiconductor. As shown in Fig. 6, it is expected that the injection of the electrons of OH^−^ to the valence band of Cu_3_Mo_2_O_9_ is more favorable compared to that of CuMoO_4_ because of its lower *E*_V_, and consequently, a higher OER activity than CuMoO_4_. Fig. 7a shows the comparative LSVs of both samples for OER measured in 0.1 M NaOH solution at 25 °C; they indicate close onset potentials. The dashed line shows the standard potential of OER. As Fig. 7a shows, OER overpotentials of 0.28 and 0.42 V at a current density of 1 mA cm^−2^ were obtained for nanostructured Cu_3_Mo_2_O_9_ and CuMoO_4_, respectively. These results are in agreement with the diagram of Fig. 6. In addition, these numerical values are in good agreement with the results of Gou *et al.*, who reported an overpotential of 0.325 mV at 50 mA cm^−2^ in 1.0 M KOH for Cu_3_Mo_2_O_9_ nanosheet loaded on nickel foam.^90^

The kinetics of OER at electrode interfaces were reflected by their corresponding polarization curves. The linear curve fitting gives the Tafel slopes of 46.9 and 79.9 mV dec^−1^ for, respectively, CuMoO_4_ and Cu_3_Mo_2_O_9_, as illustrated in Fig. 7b. These slopes are less than that of Pt/C (99 mV dec^−1^), indicating a more rapid kinetics for OER on the surfaces of these electrocatalysts compared to Pt/C.^91^

**Scheme 1:**
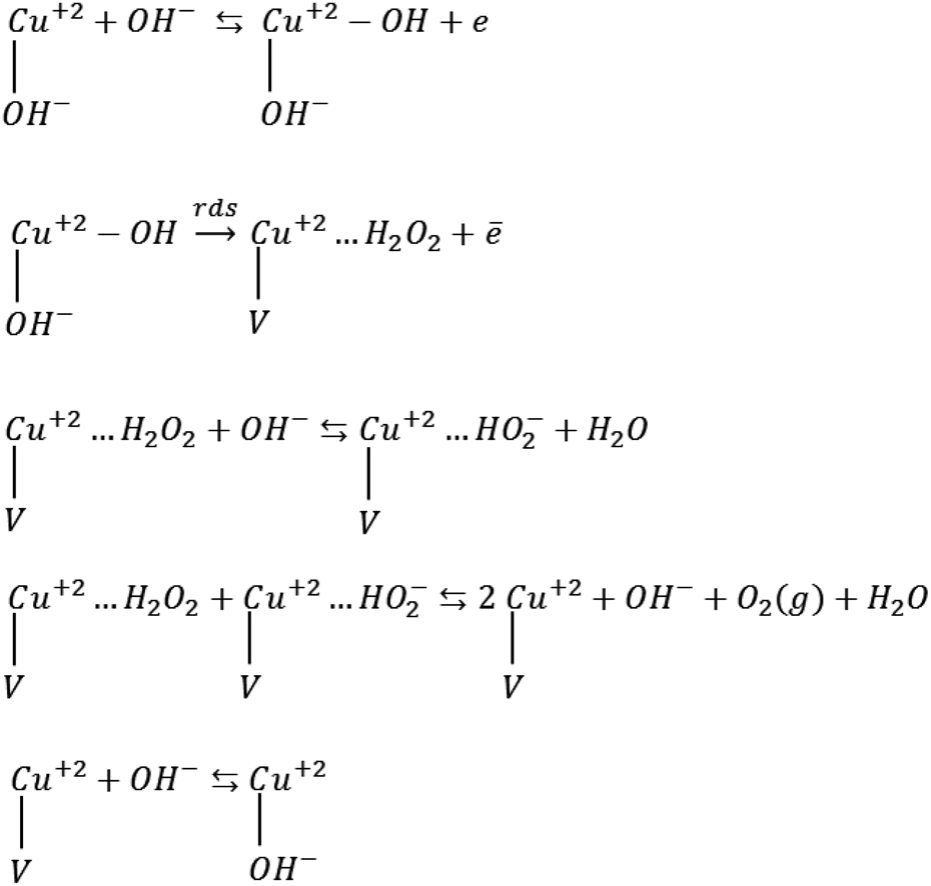
OER mechanism by emphasizing on the electrochemical desorption of OH^−^ as the rate-determining step (rds). V denotes an OH^−^ vacancy and dots show physisorbed species.

The obtained Tafel slopes are close to those reported for some nickelates and cobaltates by Bockris and Otagawa,^92^ who investigated OER on the surface of different perovskites and developed a common mechanism for OER by emphasizing on electrochemical adsorption of OH^−^ ions followed by desorption of OH and formation of H_2_O_2_ as an intermediate that is decomposed into O_2_. Such a mechanism has also been suggested for RuO_2_ (ref. 93) and TiO_2_.^94^Scheme 1 was inspired by their work.

Mott–Schottky analysis showed that Cu^2+^ ions are the localized charges in the space charge layer, thus they should be involved in interfacial phenomena. Accordingly, for the adapted mechanism, we supposed that the adsorption of OH^−^ ions occurs on the copper ions. The first step in Scheme 1 is the formation of the adsorbed OH radicals, which can occur *via* discharging of both hydroxyl ions and H_2_O molecules. However, in alkaline solution, hydroxyl ions reach the surface of electrodes faster than water molecules because of their diffusion coefficient.^95,96^ The second step, which is rds, includes the electrochemical desorption of OH radicals, the formation of physically adsorbed hydrogen peroxide and OH^−^ vacancy on the surface of these compounds. The next two steps are fast steps and show desorption and decomposition of hydrogen peroxide and the formation of O_2_ molecules. The final fast step is surface diffusion of OH^−^ into the oxygen vacancy sites. The exchange current densities (*i*_0_) (measured by extrapolation of the Tafel plot) were calculated, and the values of 6.8 × 10^−10^ and 3.1 × 10^−9^ mA cm^−2^ were obtained for CuMoO_4_ and Cu_3_Mo_2_O_9_, respectively. Generally, the exchange current density is expected to be proportional to the surface density of catalytically active sites; here, Cu^2+^ ions correspond to the below mechanism.

**Fig. 8:**
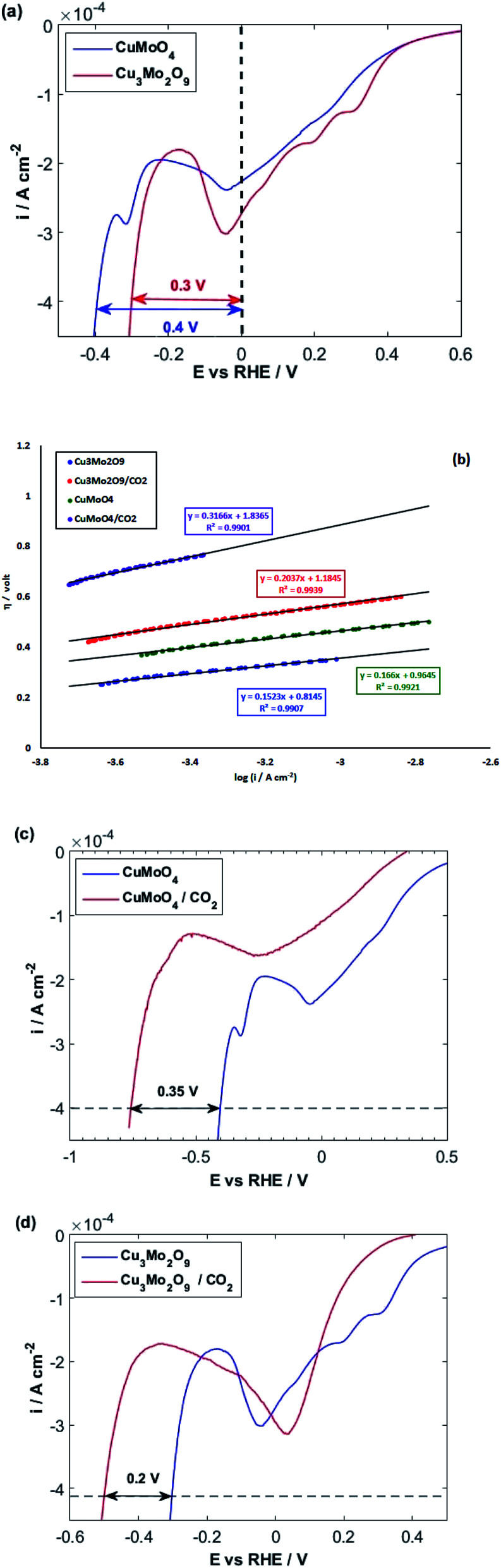
(a) LSVs and (b) linear parts of polarization curves of CuMoO_4_ and Cu_3_Mo_2_O_9_ for OER with a scan rate of 10 mV s^−1^ in 0.1 M NaOH and (c and d) polarization curves of CuMoO_4_ and Cu_3_Mo_2_O_9_ in the presence/absence of CO_2_.

After investigating the OER activity, the electrocatalytic performances of the samples were comparatively studied in terms of HER in 0.1 M NaOH solution at 25 °C. For this purpose, the LSVs of both samples were taken by starting from positive potentials to negative potentials up to the occurrence of HER, which is identified by a sharp increase in cathodic current. The resulting LSVs are depicted in Fig. 8a. It is observed that some cathodic peaks exist before hydrogen evolution. Liu *et al.* showed that the redox reaction of Mo in copper molybdate does not occur during electrochemical measurement, so the redox behavior of Mo has no contribution to the measured capacitance.^97^ Moreover, our previous study on the comparative electrochemical behaviors of CuWO_4_ and CuO showed that these peaks can be attributed to Cu(ii)/Cu(i) and Cu(i)/Cu(0) redox reactions.^64^ Cyclic voltammograms (Fig. S7†) show more detail on these redox reactions for both samples. These cyclic voltammograms clearly show that the reduction of copper ions to copper atoms occurs before HER. Therefore, HER occurs on the surface of molybdate-derived copper atoms. In fact, HER takes place in acidic media more facile than in alkaline solution. However, using acidic electrolyte has some disadvantages, such as the corrosion of the electrolyzer.^98^

Moreover, the reaction needs more energy to produce proton by O–H bond cleavage of water molecules in alkaline solution.^99^ The elementary steps for HER in alkaline solution accompanied by their Tafel slopes are:

Volmer step (120 mV dec^−1^):

8 H_2_O + M + e → M–H + OH^−^

Heyrovsky step (40 mV dec^−1^):

9 H_2_O + M–H + e → M + H_2_ + OH^−^

Tafel step (30 mV dec^−1^):

10 M–H + M–H → 2M + H_2_

Based on these steps, two mechanisms of Volmer–Heyrovsky and Volmer-Tafel are possible. The Volmer step, which is the water dissociation step, is common in both mechanisms. The energy required for this step can be provided by the adsorption of water molecules on the surface of the electrocatalyst.^100^ The stronger the water molecules adsorption the more the energy provided for the Volmer step. In addition, adsorption of OH^−^ ions on the active sites has a poisoning effect and increases the overpotential by lowering the activity because of limiting the number of sites in alkaline solution. The hydrogen binding energy is another important factor that governs the HER activity of the catalysts. In fact, a volcano-type correlation between HER exchange current densities and H-binding energy values was demonstrated, which was supported by both DFT and experimental studies.^101,102^ Furthermore, the crystallographic planes of the catalysts^103^ and the existence of an active component for water dissociation^104^ can affect the kinetics of the reaction.

As illustrated in Fig. 8a, the HER overpotential on the surface of Cu_3_Mo_2_O_9_ has a value of around 0.1 V, which is smaller than that of CuMoO_4_ at a current density of 0.4 mA cm^−2^. Because the adsorbed molecules (here, water) and the active sites (here, copper atoms) are the same in nature, the predominant factor should be the number of active sites. It is clear that the surface density of copper atoms on the surface of Cu_3_Mo_2_O_9_ is larger than that of CuMoO_4_, so more surface sites are available for HER on the surface of Cu_3_Mo_2_O_9_; this lowers the overpotential. Moreover, although a precise look indicates copper active sites, copper atoms have different arrangements and different sublayers. Reduction of copper ions to copper occurs on the surface layer of copper molybdates, and the oxygen species, which exist in the sublayers, facilitate water molecules adsorption, causing HER promotion.^105,106^ Our previous work on the comparative studies of CuWO_4_ and CuO showed that the sublayer atoms have a considerable effect on lowering HER overpotentials.^64^ The effects of the nature of sublayer atoms and their configurations on reducing the HER overpotential on the surface of copper were already reported by Zhang *et al.*; they investigated full water splitting on sulfur-doped copper oxide in comparison with metallic copper.^107^

Fig. 8b shows a Tafel slope close to 120 mV dec^−1^ for both samples highlighting that the Volmer step is the rate-determining step in HER mechanism for both samples. The Tafel slopes of 166 and 152 mV dec^−1^ were obtained, and accordingly, exchange current densities of 1.5 × 10^−6^ and 4.5 × 10^−6^ A cm^−2^ were calculated for CuMoO_4_ and Cu_3_Mo_2_O_9_, respectively. It means that the rate of HER on the surface of Cu_3_Mo_2_O_9_ is three times faster compared to CuMoO_4_, which can be expected because of the more accessible active sites of this compound. The number of surface sites was also used for identifying the smaller Tafel slope for HER on the surface of MoS_2_.^108,109^ Additionally, these results are in agreement with our previous findings that compared CuWO_4_ and CuO.^64^ We showed that exchange current density for HER on the surface of CuO is much larger than that of CuWO_4_. Similarly, as shown in Fig. 1, the structural difference between CuMoO_4_ and Cu_3_Mo_2_O_9_ is that the latter has one CuO unit for each CuMoO_4_ pair, *i.e.*, Cu_3_Mo_2_O_9_ ≡ 2CuMoO_4_ + CuO, and because of the existing CuO unit, Cu_3_Mo_2_O_9_ has a larger exchange current density.

At the final step of our electrochemical studies, we investigated the ability of both samples for CO_2_ reduction. Therefore, we purged the 0.1 M NaOH solution with CO_2_ for at least 30 min. The pH of the solution decreased from 13 to around 7 because of the following equilibrium:

11 OH^−^(aq) + CO_2_(aq) ⇆ HCO_3_^−^(aq)

12 OH^−^(aq) + HCO_3_^−^(aq) ⇆ CO_3_^2−^(aq)

At ambient temperature and natural pH, the major species inside the solution is HCO_3_^−^.^110^Fig. 8c and d illustrate that HER takes place in more negative potentials when CO_2_ is bubbled into the solution, and consequently, CO_2_ and its related species HCO_3_^−^ and CO_3_^2−^ are present in the solution, *i.e.*, the HER overpotential increases for both samples.

For investigating this process in more detail, we compared the cyclic voltammograms of both compounds with and without CO_2_ bubbling. The results are presented in Fig. S8.†

Both cathodic and anodic peaks of the samples in CO_2_ saturated 0.1 M NaOH solution show a significant decrease in height. This indicates that the adsorption of CO_2_ and maybe its reduction intermediates occur on the same sites where HER occurs. Furthermore, the adsorption of CO_2_ has a poisoning effect on HER and inhibits it.

In fact, there is a competition between H_2_O and CO_2_ molecules to be reduced on the surface of copper atoms.^111,112^ However, according to Fig. 8c and d, when CO_2_ is blown into the NaOH solution, the increasing of HER overpotential for CuMoO_4_ is more than that of Cu_3_Mo_2_O_9_ by around 0.15 V.

It means that the dissolved CO_2_ species in the solution or the adsorbed CO_2_ reduction intermediates occupy more HER active sites on the surface of CuMoO_4_ compared to Cu_3_Mo_2_O_9_. This implies the fact that the density of active sites (here, copper atoms) on the surface of Cu_3_Mo_2_O_9_ is higher compared to CuMoO_4_. Moreover, the atoms and their configuration in the sublayer have a considerable effect on the adsorption of CO_2_.

The Tafel slope analysis for HER in the presence of CO_2_ shows the same value of exchange current density of 1.5 × 10^−6^ A cm^−2^, for both samples, whereas Cu_3_Mo_2_O_9_ has a larger surface density of active sites for HER. Actually, it is possible that when HER starts on Cu_3_Mo_2_O_9_, some sites have still been occupied by CO_2_ reduction species. According to our previous report,^64^ which showed that, in the presence of CO_2_-species in the solution, HER occurs more readily than CuWO_4_. Therefore, it is possible here that HER occurs on the surface of copper atoms, which have come from CuO unit of Cu_3_Mo_2_O_9_, while CO_2_-species involved in the reaction on the surface of copper atoms have originated from CuMoO_4_ units of Cu_3_Mo_2_O_9_. This means that the adsorption of CO_2_-species is strong on the surface of CuMoO_4_.

In fact, the existence of oxygen in sublayers causes the strong adsorption of CO_2_, which will produce carbon-rich CO_2_-reduction products such as alcohols, carboxylic acids and hydrocarbons instead of CO. This conclusion can be supported by other reports. For instance, it has been reported that oxide-derived copper produces methanol,^113–115^ ethylene and ethanol with increased current efficiency.^116^ Moreover, the existence of tin oxide layer over tin effectively produces formate^117,118^ and formic acid.^119^ Tayyebi *et al.* used DFT calculation for electrochemical reduction of CO_2_ for producing methanol, methane and formic acid over different transition metal oxides, and showed that there is a volcano-shaped behavior through the scaling relations of adsorbed intermediates.^120^

## 4. Conclusion

In this study, two types of copper molybdate, CuMoO_4_ and Cu_3_Mo_2_O_9_, were synthesized successfully in nanoscale. Our results showed that the difference between their electrochemical behaviors strongly depends on their crystal structure. In fact, the existence and addition of CuO unit in the unit cell of Cu_3_Mo_2_O_9_ caused a modified behavior corresponding to a combination of CuO and CuMoO_4_.

The value of the Tafel slope for Cu_3_Mo_2_O_9_ (as OER) was much lower than that for CuMoO_4_. Therefore, Cu_3_Mo_2_O_9_ shows a better catalytic performance for both HER and OER in 0.1 M alkaline solution. The literature review indicated that Cu_3_Mo_2_O_9_ could be a bifunctional water splitting catalyst under alkaline conditions, and show better activity at low cell voltage than CuMoO_4_. The superior OER and HER activities for Cu_3_Mo_2_O_9_ can be attributed to the more active sites arising from oxygen vacancies or the unique topology of Cu_3_Mo_2_O_9_ crystals that maximizes the number of exposed active sites. However, our investigation showed that CO_2_-species are adsorbed on the surface of CuMoO_4_ more strongly than on the surface of Cu_3_Mo_2_O_9_, so they may produce more stable and carbon-rich products.

## Conflicts of interest

There are no conflicts to declare.

## Supplementary Material

RA-010-D0RA07783F-s001
