# Supplementary material for: Nanostructured copper molybdates as promising bifunctional electrocatalysts for overall water splitting and CO2 reduction
Source: RSC Adv. 2020 Oct 23;10(64):39037–48. doi: 10.1039/d0ra07783f (PMC9057328; doi:10.1039/d0ra07783f)
Supplement: RA-010-D0RA07783F-s001 [file RA-010-D0RA07783F-s001.pdf]

Electronic Supplementary Material (ESI) for RSC Advances.
This journal is © The Royal Society of Chemistry 2020

Electronic Supplementary information

Nanostructured copper molybdates as promising bifunctional electrocatalysts for overall water splitting and CO_2 reduction

Atefeh Rahmani[1], Hossein Farsi[*1,2]

1) *Department of Chemistry, University of Birjand, Birjand, Iran*
2) *Developing Nanomaterials for Environmental Protection Research Lab, University of Birjand, Birjand, Iran*

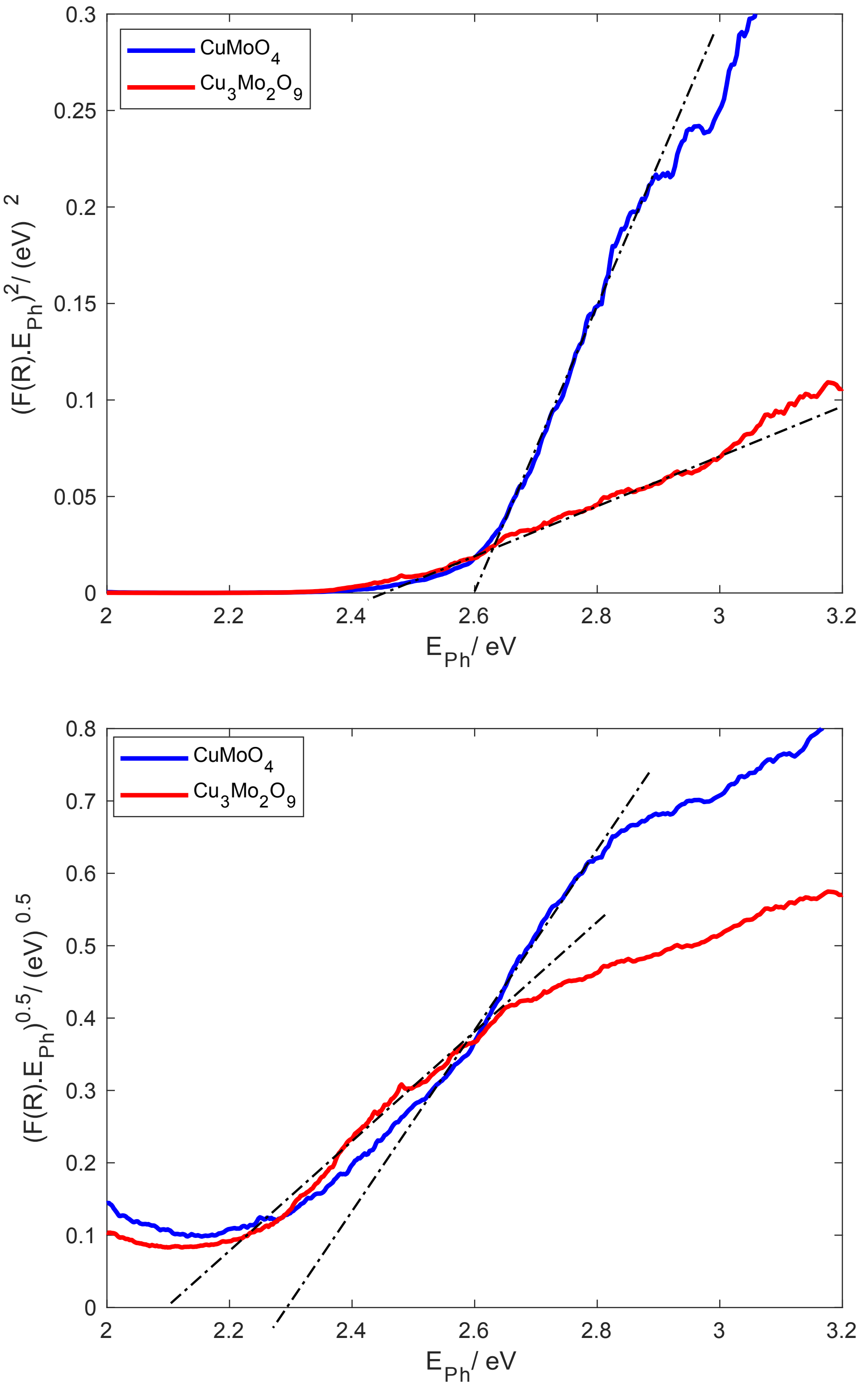

Fig. S1. Transformed DRS data for a) direct and b) indirect transitions.

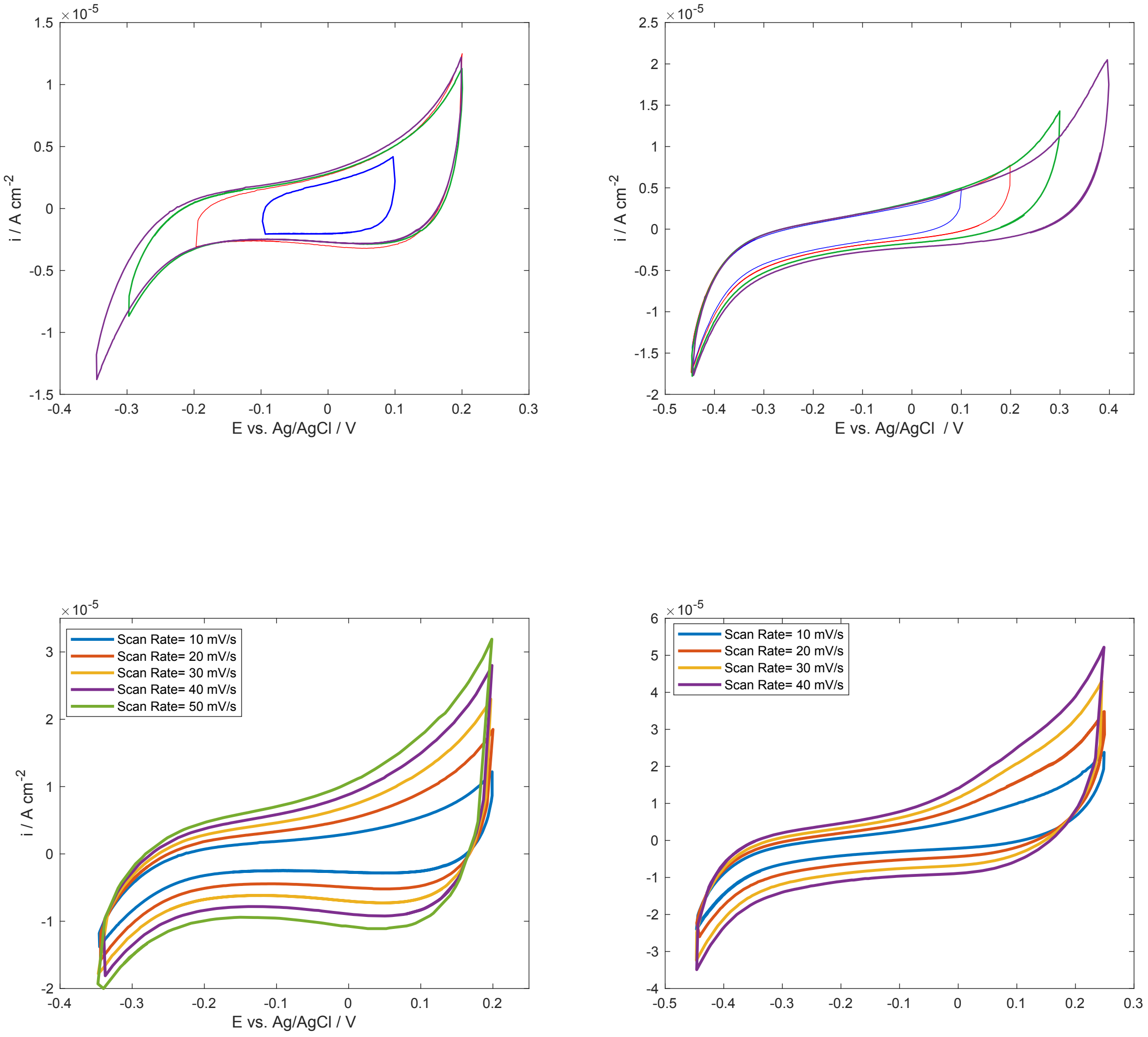

Fig. S2. Cyclic voltammograms of a, c) $CuMoO_4$ and b, d) $Cu_3Mo_2O_9$ at different end potentials and scan rates in 0.1 M NaOH.

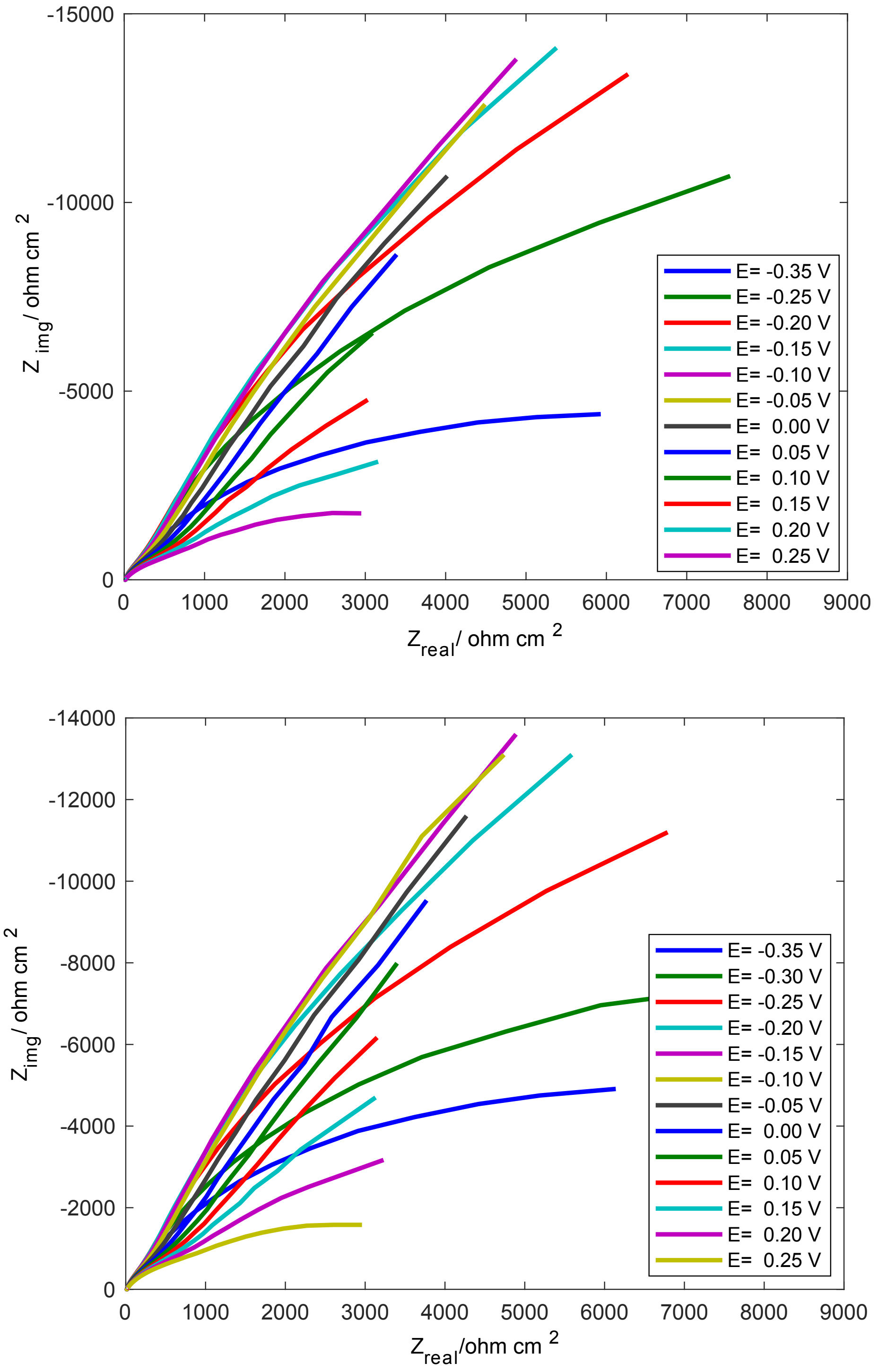

Fig. S3. Nyquist diagrams of nano-$CuMoO_4$ in 0.1 M NaOH for a) in the dark and b) under illumination.

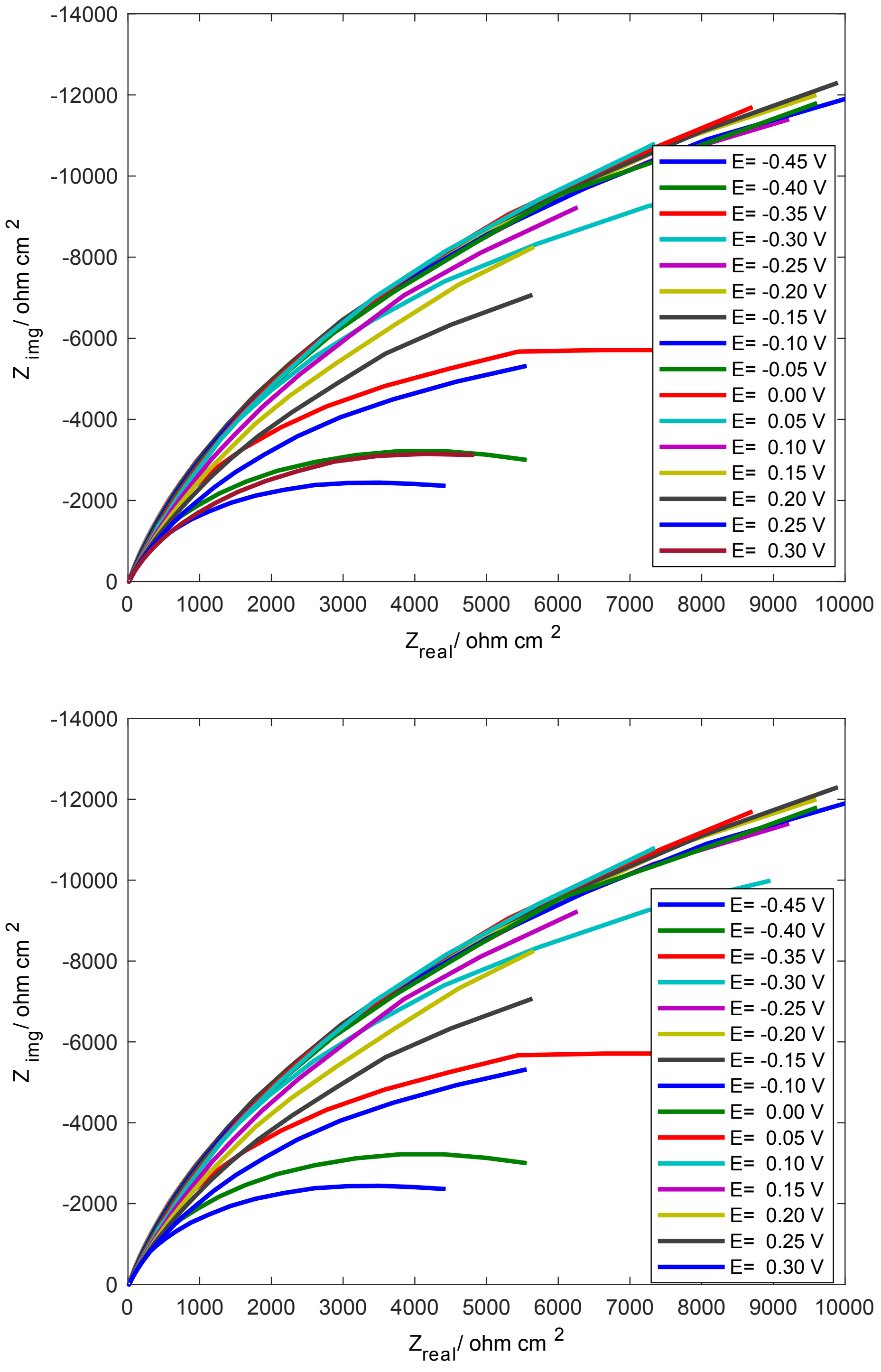

Fig. S4. Nyquist diagrams of nano-$Cu_3Mo_2O_9$ in 0.1 M NaOH for a) in the dark and b) under illumination.

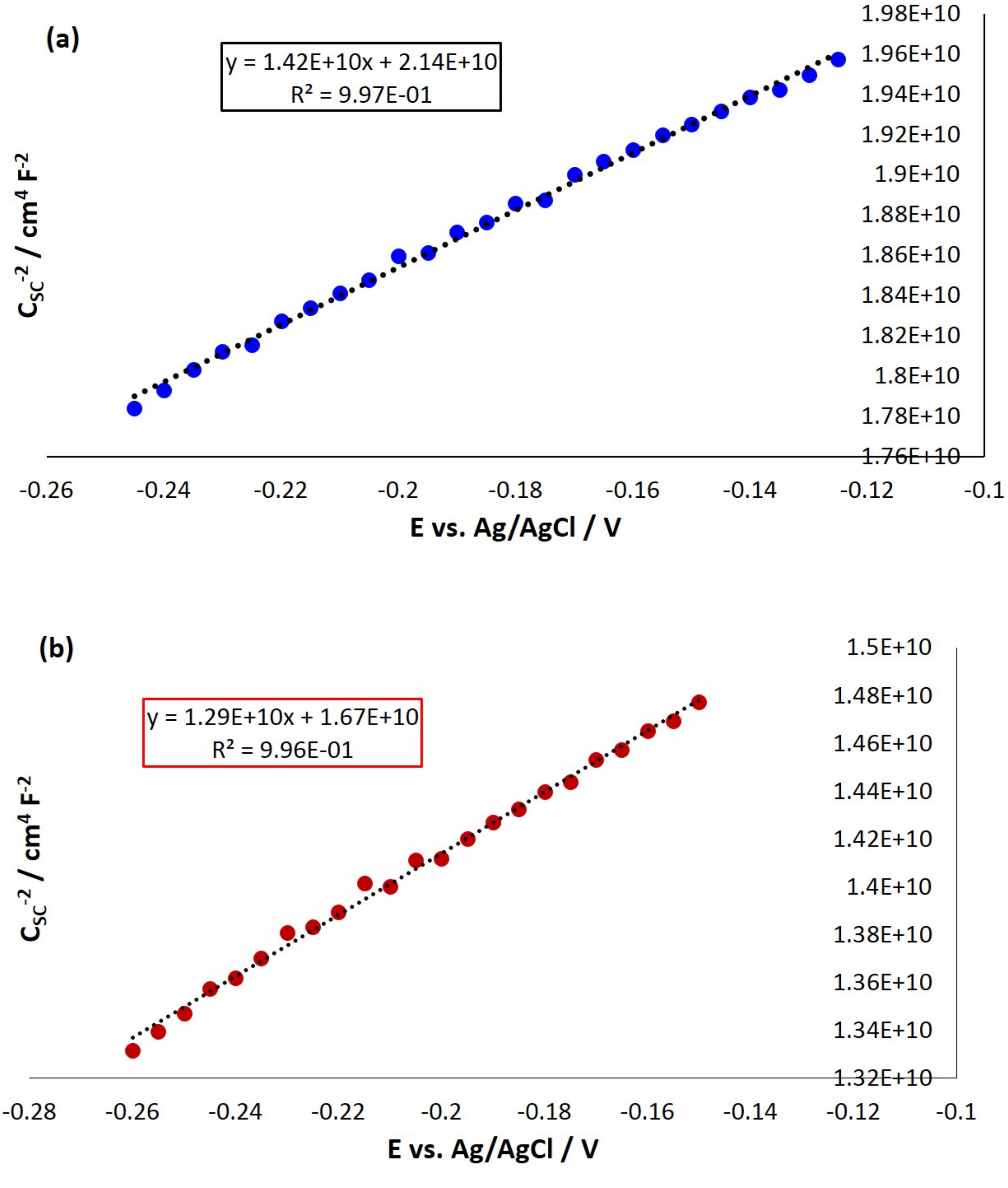

Fig. S5. Linear part of Mott-Schottky plot of nano-$CuMoO_4$ in 0.1 M NaOH for a) in the dark and b) under illumination.

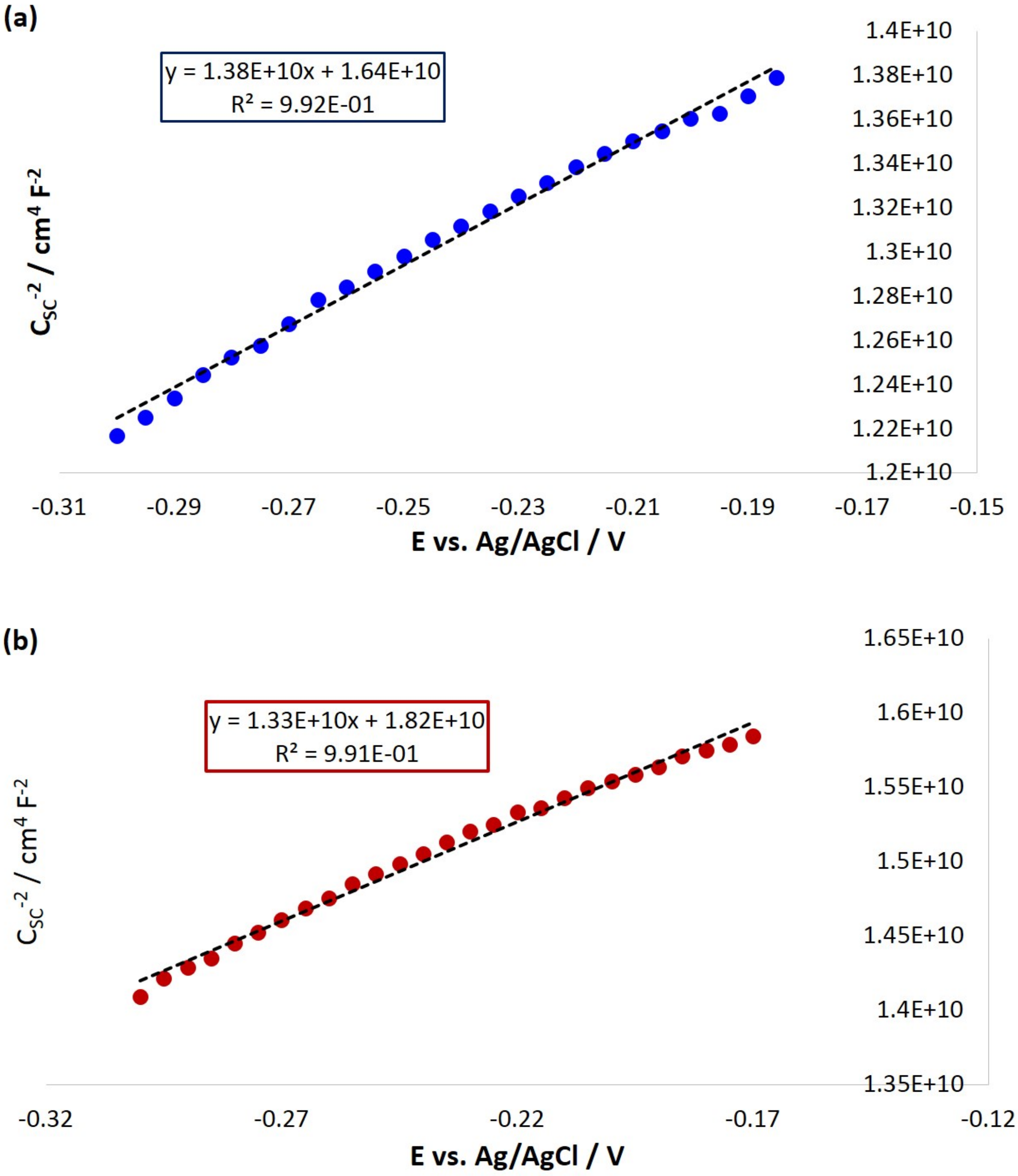

Fig. S6. Linear Mott-Schottky plot of nano-$Cu_3Mo_2O_9$ in 0.1 M NaOH for a) in the dark and b) under illumination.

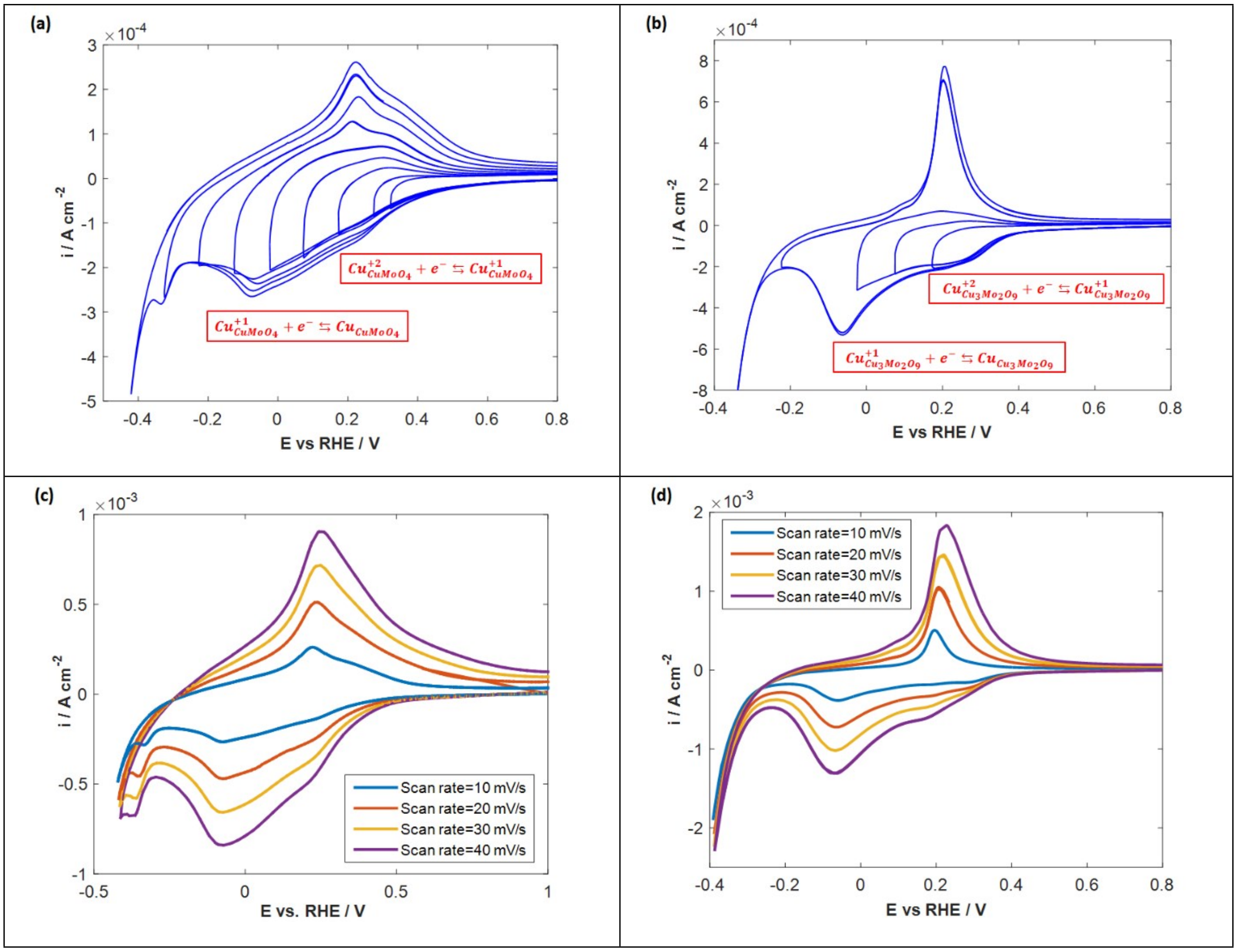

Fig. S7. Cyclic voltammograms with different end potentials for a) $CuMoO_4$ and b) $Cu_3Mo_2O_9$, at different scan rates for c) $CuMoO_4$ and d) $Cu_3Mo_2O_9$.

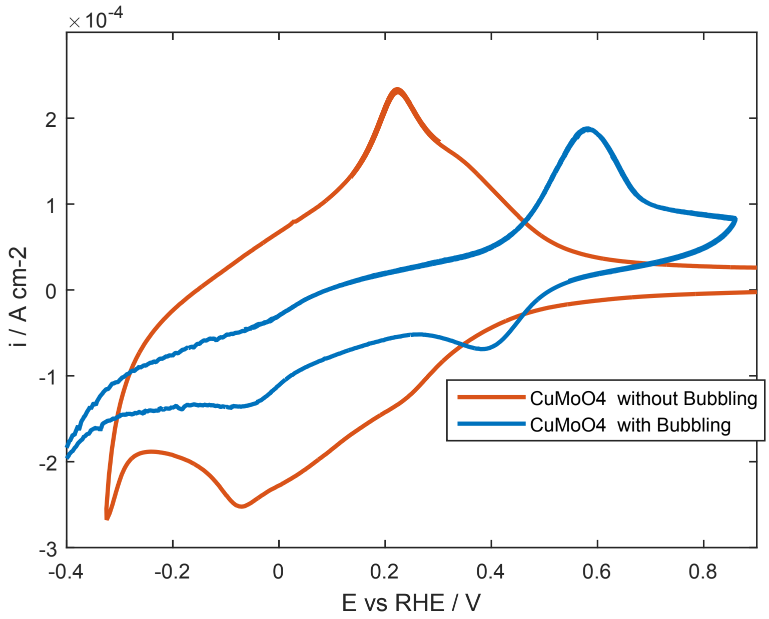

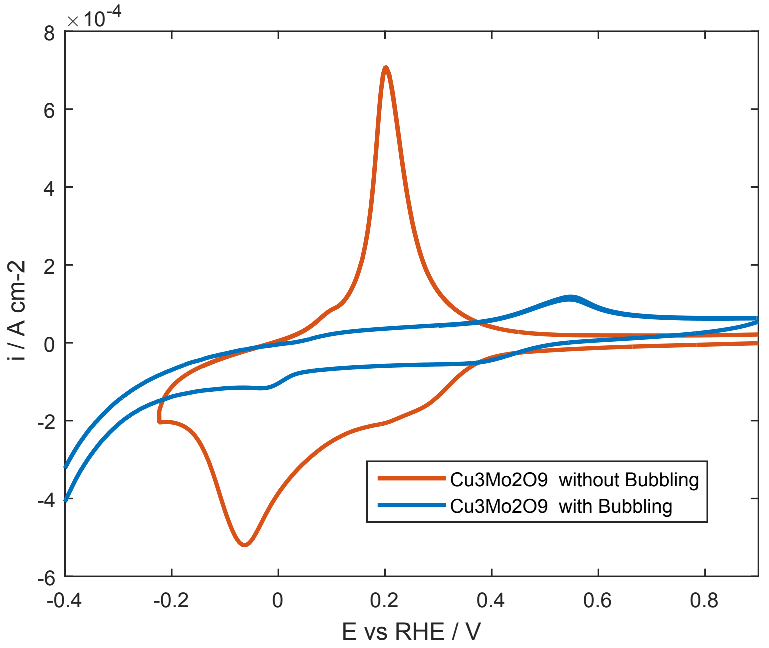

Fig. S8. Comparative CVs of a) $CuMoO_4$, and b) $Cu_3Mo_2O_9$ in the presence and absence of CO_2 dissolving species.
